# The impact of Covid-19 pandemic on hospices: A systematic integrated review and synthesis of recommendations for policy and practice

**DOI:** 10.12688/amrcopenres.13105.1

**Published:** 2022-10-26

**Authors:** Shalene van Langen-Datta, Helen Wesson, Joanna Fleming, Abi Eccles, Catherine Grimley, Jeremy Dale, Kathryn Almack, Catriona Mayland, Sarah Mitchell, Ruth Driscoll, Lynn Tatnell, Lesley Roberts, John I. MacArtney

**Affiliations:** 1 Marie Curie, London, SE1 7TP, UK; 2 Unit of Academic Primary Care, Warwick Medical School, University of Warwick, Coventry, Warwickshire, CV4 7AL, UK; 3 University of Hertfordshire, Hatfield, Hertfordshire, AL10 9AB, UK; 4 Department of Oncology and Metabolism, University of Sheffield, Sheffield, S10 2TN, UK; 5 Patient or Public Involvement (PPI) Representative, University of Warwick, Coventry, CV4 7AL, UK

**Keywords:** Covid-19; hospice; specialist palliative care; policy; integrated review

## Abstract

**Background:**

The Covid-19 pandemic resulted in the development of numerous recommendations for practice and policy for specialist palliative care provided by hospices in United Kingdom (UK), as hospices were significantly affected by the pandemic and protections put in place.

The aim of this review is to identify and synthesise recommendations or implications for policy and practice that have been generated for adult hospice specialist palliative care during the first 24 months of the Covid-19 pandemic.

**Methods:**

AMED, BNI, CINAHL, EMBASE, EMCARE, HMIC, Medline, PsycINFO, PubMed databases were searched for peer-reviewed papers, as well as hand searchers for grey literature. Literature relating to hospices and Covid-19 in the UK were included and a thematic synthesis of recommendations for hospice policy and practice was undertaken.

**Results:**

858 articles were identified with 12 meeting the inclusion criteria. Fifty-eight recommendations or implications were identified: 31 for policy, 27 for practice, and 10 covering both. Recommendations were organised under ten themes. There were several recommendations seeking to secure hospice resources to mitigate the short-term impact of the pandemic, as well as those focused on longer-term implications such as core funding. The impact of the pandemic on the quality of hospice care was the focus for numerous recommendations around improving integration of hospice care in the community, provision of bereavement support and better use of Advance Care Plans (ACP). However, there were significant gaps related to carer visitation in hospices, inequities of palliative care, or hospice-at-home services.

**Conclusion:**

The Covid-19 pandemic and protections exposed several ongoing policy and practice needs, especially around hospice resources, while generating novel issues for hospices to address. Significant policy gaps remain to be addressed to mitigate the impact of the pandemic on the quality of hospice specialist palliative care.

## Plain language summary

Hospices in the UK faced many challenges during the first two-years of the Covid-19 pandemic. In this time several research studies and reviews took place that provided hospices with recommendations for how to adapt their policies and clinical practices. In this review we identified 12 documents that contained 58 recommendations for hospices’ policy and practice.

We grouped these recommendations together under ten key themes. We found that there were several recommendations aiming to secure hospice resources to mitigate the short and longer-term impacts upon hospice funding. The impact of the pandemic on the quality of hospice care was the focus for numerous recommendations around improving integration of hospice care in the community, provision of bereavement support and better use of Advance Care Plans (ACP). However, there were significant gaps related to carer visitation in hospices, inequities of palliative care, or hospice-at-home services.

## Background

In March 2020, healthcare services in the UK experienced an unprecedented upheaval in how they were expected to operate with the implementation of a nationwide lockdown along with other health and social protections ( Coronavirus Act, 2020). Healthcare guidance, practices, and routines that had been established over the preceding decades were brought into question in light of this new strategic and operational context ( Atkinson *et al.*, 2020). Moreover, this pandemic context necessitated quick implementation of new ways of working ( Dunleavy *et al.*, 2021).

Situated within this wider healthcare upheaval of policy and practice were hospices. Although grouped under one description, hospices are a diverse body of healthcare organisations. Most are independent charities (n=192; Hospice UK, 2021), so stand apart from the UK’s National Health Service (NHS), with government funding averaging 32% (range 20-50%) of expenditure ( Hospice UK, 2021). However, similar to the NHS, most hospices are committed to providing free-at-the-point-of-use care and support. Their services are aimed at people with life-limiting or terminal conditions and those that care for them – such as friends and family – and who have complex palliative care needs ( Clark, 2014a). The range of services each hospice provides will differ, but can include in-patient care, day, drop-in, or wellbeing services, specialist out-patient clinics, home visits and bereavement support. Hospices can be consultant or nurse led, and usually provide some combination of multi-disciplinary support from occupational therapists, physiotherapists, psychologists, social workers, spiritual support ( NCPC *et al.,* 2015).

The people who attend and use hospice services are likely to have conditions that make them some of the most at risk from a Covid-19 infection leading to death ( Treskova-Schwarzbach *et al.*, 2021), as well as it severely affecting the quality of their life. Hospice care is predicated on valuing the life a person has left and ensuring they receive holistic (physical, emotional, social and spiritual) support ( Clark, 2014b). The circulation of Covid-19 and the pandemic protections presented a double bind for hospices, as they sought ways to maintain their ‘gold-standards’ of care ( Calanzani *et al.,* 2013), while also fearful that the provision of that support might rise the risks of transmitting Covid-19. This conundrum was multiplied by the range of services that hospices seek to provide as part of their holistic offering, as well as the numerous locales in which that support can be provided.

Hospices therefore needed to rapidly modify, transform and even invent new services to support people with life-limiting conditions and those that care for them. During the first two years of the pandemic, a number of research studies explored aspects of how the pandemic affected the care of people with life-limiting conditions, leading to a range of implications and recommendations for policy and practice. However, there has been no attempt to collate or synthesise this growing body of recommendations nor establish what areas remain in need of intervention.

This scope of this review was developed as part of a wider research study that sought to explore the impact of Covid-19 pandemic on hospices for an adult population, with the aim of producing recommendations for policy and practice. This review was developed so that such recommendations could be contextualised with the rapidly evolving policy landscape.

### Aim

The aim of this review was to identify and synthesise recommendations and implications for policy and practice that have been generated for adult hospice specialist palliative care during the first 24 months of the Covid-19 pandemic.

## Methods

### Design

We undertook a systematic integrated review, an approach that allows for summarising and synthesising qualitative findings ( Seers, 2012). The review is reported in line with relevant items on the Preferred Reporting Items for Systematic Reviews and Meta-Analyses (PRISMA) checklist ( Page *et al.*, 2021) ( MacArtney, 2022).

### Search strategy

In March 2022 we searched the existing literature, using “((hospice care OR end of life care OR terminal care) AND (COVID-19 OR coronavirus)).ti,ab” in the following databases: AMED, BNI, CINAHL, EMBASE, EMCARE, HMIC, Medline, PsycINFO, PubMed. Grey literature was also searched for any charity or other healthcare sector reports eligible for inclusion, along with reference sections for any included articles were screened for potential studies to be included.

### Inclusion and exclusion criteria

A set of inclusion and exclusion criteria were developed prior to the searches, which limited the eligible papers to those reporting on the impact of the Covid-19 pandemic on hospice services in the UK ( Table 1).

**Table 1.** Inclusion and exclusion criteria.

Inclusion	Exclusion
Studies exploring the impact or effects of the pandemic on adult hospice specialist palliative care in the UK	Did not include hospices in their sample.
Studies published in English	Studies reporting wholly outside of the UK healthcare context
Studies published between 1 March 2020 and 28 February 2022.	Studies not related to the Covid-19 pandemic
	Participants identified as children or <18 years old

### Screening and data extraction

Duplicates were removed and titles and an initial sift of abstracts was undertaken to check for eligibility by SLD. The title and abstracts of the remaining articles were then read and considered for inclusion by SLD, with any queries discussed with JM. A sub-set of articles were also checked by JM to ensure accuracy. Relevant data, including any recommendations or implications for policy or practice, were extracted by SLD into the Data Extraction Table ( Table 2), and checked by HW. The information extracted from each article was: author(s) year of publication; methods and participants; recommendations or implications for policy or clinical practice ( Table 2).

**Table 2.** Data extraction table.

	Article	Aims	Study design/setting	Participants	Recommendations or implications
1.	Better End of life 2021, Marie Curie.	Better End of Life project aimed to explore the impact of COVID-19 on death, dying and bereavement in the UK (looking specifically at experience, mortality changes/ places of death, the ongoing role of specialist palliative care and contribution of services to wider COVID-19 effort, role of primary care in ongoing palliative care, challenges faced by those bereaved during the pandemic, make recommendation according to findings re. ongoing pandemic hospices (health and social care policy).	Patients, families, published literature, secondary analysis of data, analysis of publicly available data.	Section 2 of the report re. carers includes 3 perspectives (section 3–5 rapid evidence review and data/no participants). Sections 6–7: based on CovPall study (total 458 services surveyed, of which 277 services were in the UK). Section 8: Mitchell and Mayland study data: 559 participants (387 community nurses, 156 GPs, 16 unspecified). Section 9 (Harrop et al bereavement experience study, 532 participants, Pearce and Barclay study, 805 practitioners e.g. hospice social workers, palliative medicine doctor, hospice services manager). Section 10 – a PPI member’s reflection.	1. Specialist palliative care services within and outside of the NHS must be resourced appropriately 2. Palliative care services have played a front line role during COVID 19 and this role must be recognised 3. Societal preferences and expectation for death and dying have permanently changed and the health and social care system must respond accordingly 4. Care homes must be recognised as important providers of palliative and end of life care and supported appropriately 5. Family members and carers must be recognised as important providers of palliative and end of life care and supported 6. Primary care services need to be recognised as increasingly important providers of palliative and end of life care, at home and in care homes 7. Increased provision of bereavement services is urgently needed 8. Data systems must include information on dying death and bereavement 9. Research that informs new care for people affected by dying, death and bereavement during Covid-19 is urgently required 10. The UK government should work with local authorities to introduce a fast-track passport for people who have a diagnosed terminal illness that entitles them to access all relevant benefits and services on a fast-track or priority basis.
2.	Bayly, J., Bradshaw, A., Fettes, L., Omarjee, M., Talbot-Rice, H., Walshe, C., ... & Maddocks, M. (2021). Understanding the impact of the Covid-19 pandemic on delivery of rehabilitation in specialist palliative care services: An analysis of the CovPall-Rehab survey data. *medRxiv* .	To understand rehabilitation provision in palliative care services during the Covid-19 pandemic, identifying and reflecting on adaptative and innovative practice to inform ongoing provision.	Cross-sectional national online survey, conducted from 30/07/20 to 21/09/2020.	61 rehabilitation leads for specialist palliative care services across hospice, hospital, or community settings	11. Recommendations are made to support extended reach and more equitable access to rehabilitation in palliative care services. 12. We recommend mixed methods evaluations of hybrid models of in-person and online rehabilitation across palliative care settings.
3.	Dunleavy, L., Preston, N., Bajwah, S., Bradshaw, A., Cripps, R., Fraser, L. K., ... & Walshe, C. (2021). ‘Necessity is the mother of invention’: Specialist palliative care service innovation and practice change in response to COVID-19. Results from a multinational survey (CovPall). Palliative medicine, 35(5), 814-829.	To map and understand specialist palliative care services innovations and practice changes in response to COVID-19.	Online survey of specialist palliative care providers (CovPall), disseminated via key stakeholders. Data collected on service characteristics, innovations and changes in response to COVID-19.	458 inpatient palliative care units, home nursing services, hospital and home palliative care teams from any country. 277 UK, 85 Europe (except UK), 95 World (except UK and Europe), 1 missing country. 54.8% provided care across 2+ settings; 47.4% hospital palliative care teams, 57% in-patient palliative care units and 57% home palliative care teams.	13. In addition to financial support, greater collaboration is essential to build organisational resilience and drive forward innovation, by minimising duplication of effort and optimising resource use. 14. The effectiveness and sustainability of any changes made during the crisis needs further evaluation
4.	Pearce, C., Honey, J. R., Lovick, R., Creamer, N. Z., Henry, C., Langford, A., ... & Barclay, S. (2021). ‘A silent epidemic of grief’: a survey of bereavement care provision in the UK and Ireland during the COVID- 19 pandemic. BMJ open, 11(3), e046872.	To investigate the experiences and views of practitioners in the UK and Ireland concerning changes in bereavement care during the COVID-19 pandemic.	Online survey using a snowball sampling approach. Practitioners working in hospitals, hospices, care homes and community settings across the UK and Ireland.	Health and social care professionals involved in bereavement support. 805 respondents working in hospice, community, and hospital settings across the UK and Ireland completed the survey between 3 August and 4 September 2020.	15. Improved resources for existing bereavement services to enable coordination between local, regional and national networks, and encourage a sustainable model of bereavement care. 16. Developing a proactive approach to supporting those bereaved during this period and making services accessible for all. 17. Enabling regular communication with families prior to a death and after to ensure families have opportunities to ask questions and receive reassurance. 18. Where possible, find ways for families to be with dying loved ones. 19. Integrating assessment of bereaved families’ needs into communication to help identify and signpost those who might require further support. 20. Training in bereavement care to be integrated into medical, nursing and other healthcare professional training. 21. Acknowledging the challenges on staff and encourage brief training for those who feel unequipped to manage needs of grieving families.
5.	Selman, L., Lapwood, S., & Jones, N. (2020). What enables or hinders people in the community to make or update advance care plans in the context of Covid-19, and how can those working in health and social care best support this process.	What enables or hinders people in the community to make or update advance care plans in the context of Covid-19? How can staff working in health and social care best support this process? The objectives are to: At present, what is known about ACP in community settings, considering relevance to people with COVID-19 report on the feasibility, acceptability, challenges/barriers and facilitators/ enablers of ACP in the context of COVID-19, where the need for infection control measures can prevent face-to-face ACP discussions summarise emerging evidence and clinical guidelines relevant to ACP in the community during the COVID-19 pandemic	Rapid evidence review with narrative synthesis of the published literature. Search strategy: English language publications on PubMed, Embase (OvidSP) [1974- present], LitCOVID, medRxiv, Google Scholar and Google up to 7/7/20. Topic broken into four main searches: Advance Care Planning/End of Life Communication during COVID-19, Interventions to improve Advance Care Planning or End of Life Communication – Systematic Reviews [2010 onwards], Telehealth and mobile technologies for Advance Care Planning or End of Life Communication [2010 onwards], Advance Care Planning or End of Life Communication and Personal Protective Equipment	21 research studies and 10 systematic reviews that met our inclusion criteria and were classed as highly relevant (see Appendix 2 for data extracted; quality appraisal results available on request). 12 guidelines related to ACP in the UK during COVID-19.	22. In the context of COVID-19, and to reduce inequalities in access to Advance Care Planning (ACP), we recommend national investment in evidence-based, public-facing resources and integrated systems to support ACP, building on existing resources. 23. Alongside this investment, simultaneous, interconnected strategies are needed, underpinned by healthcare policy: training for those working in health and social care, better coordination of electronic medical record systems, and public education and awareness raising. Those working in health and social care can support ACP in the community by: 24. Informing the public about the processes and legal status of ACP and dispelling fears and misperceptions, e.g. that ACP is related to rationing healthcare resources. 25. Creating opportunities for ACP conversations among patients and residents early, particularly among older people and those at increased risk, discussing ACP over several sessions and revisiting decisions. 26. Sign-posting to appropriate written, web-based and audio- visual ACP resources. 27. Adapting ACP to the individual and, if appropriate, including the opportunity to complete ACP documentation, without focusing on this. 28. Using remote consultations for ACP discussions where needed and appropriate, drawing on best practice guidelines. 29. Helping to cultivate a culture of openness around ACP in nursing home settings and having ongoing ACP conversations with residents (including those with cognitive impairment) and their family members. 30. Ensuring ACP discussions are fully and promptly recorded in patient records which are accessible to those who need them. Health and social care policy can support ACP in the community by: 31. Targeting multiple levels of influence (individual, interpersonal, provider, system) to ensure ACP interventions are effective, sustainable and have maximum reach during the pandemic. At present most ACP guidelines focus on clinicians. 32. Introducing coordinated and consistent public health messaging that reframes ACP as routine and normal for anyone interested in considering and influencing their future care, making ACP driven by the public and supported (rather than owned) by health and social care professionals. 33. Creating a robust, nationally co-ordinated and public-facing web portal for ACP resources to facilitate this shift and increase awareness and uptake, harnessing the increased use of technological approaches to care and communication during the pandemic. It is essential that resources are diverse, use audio-visual as well as written formats, and are designed to support disadvantaged communities. 34. Ensuring each country in the UK has a comprehensive policy to support ACP and aid its implementation, monitoring and evaluation. 35. Prioritising research into an integrated web-based system for ACP in which members of the public could create an advance care plan which links to their medical record.
6.	Oluyase, A. O., Hocaoglu, M., Cripps, R. L., Maddocks, M., Walshe, C., Fraser, L. K., ... & CovPall study team. (2021). The challenges of caring for people dying from COVID-19: a multinational, observational study (CovPall). Journal of pain and symptom management.	To understand the response of and challenges faced by palliative care services during the COVID-19 pandemic, and identify associated factors.	Survey of palliative care and hospice services, contacted via relevant organizations.	A total of 458 services responded; 277 UK, 85 rest of Europe, 95 rest of the world	36. Despite actively supporting dying patients, those with severe symptoms, their families/carers, other supporting clinicians, palliative care professionals felt ignored by national health systems during the COVID-19 pandemic. 37. Palliative care services need equipment, medicines, and adequate staff to contribute fully to the pandemic response. Their crucial role must be better recognised and integrated, including into infection disease management, with improved workforce planning and management, so that patients and families can be better supported. 38. The crucial role of palliative care during pandemics must be better recognised and integrated. This is particularly the case for charity managed services and those providing care in people’s homes. 39. Beyond COVID-19, this research has shed light on the limited integration of palliative care, the urgent need to increase its workforce and a need for palliative skills to be a core part of the training of clinicians.
7.	Hanna, J. R., Rapa, E., Dalton, L. J., Hughes, R., Quarmby, L. M., McGlinchey, T., ... & Mason, S. R. (2021). Health and social care professionals’ experiences of providing end of life care during the COVID-19 pandemic: A qualitative study. *Palliative Medicine*, 02692163211017808.	To explore health and social care professionals’ experiences of providing end of life care during the COVID-19 pandemic to help inform current/future clinical practice and policy.	A qualitative interview study. Data were analysed using thematic analysis.	16 health and social care professionals working across a range of clinical settings in supporting dying patients during the first wave (March–June 2020) of the COVID-19 pandemic in the United Kingdom.	40. Tools such as question prompt lists and charting daily family communication could help promote informative family engagement at times of restricted visiting. 41. Clarity in guidance and governance is required to identify when relatives can visit a dying family member in institutional settings during a pandemic, with a clear recommendation that this contact should be facilitated when death is expected in weeks and days rather than hour(s) before death. 42. There is a need for visible leadership and support within healthcare teams to promote self-care and reflection, as well as ongoing access to psychological support for health and social care professionals.
8.	Nestor, S., O’Tuathaigh, C., & O’Brien, T. (2021). Assessing the impact of COVID-19 on healthcare staff at a combined elderly care and specialist palliative care facility: a cross-sectional study. *Palliative* * Medicine*, *35*(8), 1492-1501.	To describe and characterise the magnitude and variety of ways in which the COVID-19 pandemic affected the personal, social and professional lives of healthcare workers representing several multidisciplinary specialties in a fully-integrated palliative and elderly care service.	Anonymised standardised questionnaire evaluating the impact of COVID-19 across a diverse range of domains. The study was conducted over a 6-week period commencing 11 September 2020. The setting incorporates two distinct but integrated services operating under a single management structure in Ireland: (i) Specialist palliative care across hospice (44 beds), community and hospitals and (ii) Elderly Care Service (long- term and respite care) delivered in a 63-bed inpatient unit.	250 respondents (69.8%) completed the questionnaire. Nurses and healthcare assistants comprised the majority of respondents (60%) and other disciplines were represented proportionately.	43. Need to recognise the importance of supporting all staff and keeping them safe by initiatives such as access to appropriate PPE; education, including support with adapting to greater use of IT; clear communication strategies, accurate and consistent information; staff involvement in protocol development and implementation, introduction of innovative means of communication with family members and colleagues
9.	Bradshaw, A., Dunleavy, L., Walshe, C., Preston, N., Cripps, R. L., Hocaoglu, M., ... & CovPall study team. (2021). Understanding and addressing challenges for advance care planning in the COVID-19 pandemic: An analysis of the UK CovPall survey data from specialist palliative care services. *Palliative* * Medicine*, 02692163211017387.	Describe the challenges that UK specialist palliative care services experienced regarding Advance Care Planning (ACP) during COVID-19 and changes made to support timely conversations.	Online survey of UK palliative/ hospice services’ response to COVID-19. Closed-ended responses are reported descriptively. Open-ended responses were analysed using a thematic Framework approach using the Social Ecological Model to understand challenges.	Two hundred and seventy-seven services, of which 168 included hospice services.	44. COVID-19 has provided an opportunity to re-think advance care planning in which the starting point to any discussion is always the values and priorities of patients themselves. 45. Providers and policymakers need to urgently consider how high-quality advance care planning can be resourced and normalised as a part of standard care across the health sector, ahead of future or recurrent pandemic waves and in routine care more generally. 46. There are several key questions that health professionals, services, and policy makers ought to consider in working towards this.
10.	Mayland, C. R., Hughes, R., Lane, S., McGlinchey, T., Donnellan, W., Bennett, K., ... & Mason, S. R. (2021). Are public health measures and individualised care compatible in the face of a pandemic? A national observational study of bereaved relatives’ experiences during the COVID-19 pandemic. *Palliative* * Medicine*, 02692163211019885.	To explore bereaved relatives’ experiences of quality of care and family support provided during the last days of life; to identify the impact of factors associated with perceived support.	A national, observational, open online survey disseminated via social media, public fora and professional networks (June– September 2020). Validated instruments and purposively designed questions assessed experiences. Analysis used descriptive statistics, logistic regression and thematic analysis of free-text responses.	Individuals (≥18 years) who had experienced the death of a relative/friend (all care settings) within the United Kingdom during the COVID-19 pandemic. 278 respondents, most ( n = 216, 78%) were female.	47. Providing staff training and enabling protected time for timely, informative communication between health and social care professionals and family members needs to be prioritised during a pandemic, especially within the care home setting where this is less commonplace. 48. During a pandemic, it is essential that health and social care staff can recognise dying and feel confident to talk honestly with relatives about this, to enable final visits to be conducted in a timely manner. 49. There is a need to identify additional elements that explain differing perceptions of support during a pandemic, to help tailor support mechanisms both before and after the death
11.	Mitchell, S., Oliver, P., Gardiner, C., Chapman, H., Khan, D., Boyd, K., ... & Mayland, C. R. (2021). Community end-of-life care during the COVID- 19 pandemic: findings of a UK primary care survey. *BJGP open*, *5*(4).	To understand the views of GPs and community nurses providing end-of- life care during the first wave of the COVID-19 pandemic.	A web-based, UK-wide questionnaire survey circulated via professional general practice and community nursing networks, during September and October 2020.	559 respondents (387 community nurses, 156 GPs, and 16 unspecified roles), from all regions of the UK	50. Opportunities and potential unintended consequences in the use of virtual technology for remote consultations with patients at the end of life and their families must be better understood if this practice is to continue. 51. The potential of technology to improve inter-professional communication requires further investigation. 52. There is an immediate need for policymakers and commissioners to recognise the sustained increased need for end-of-life care in the community and the critical role of primary healthcare services in the delivery of this care. 53. Findings suggest a disconnect between teams involved in end-of-life care in the community and a need to rebuild trusted relationships through truly integrated approaches between GPs, community nurses, and specialist palliative care services. 54. Policy guidance and service models must place focus on and support the multidisciplinary team relationships that are necessary to deliver this care most effectively. Current guidance relating to the roles of specific services has the potential to fragment teams. 55. Ensuring support for individuals involved in the provision of this care, through team relationships, training opportunities, and debrief also requires attention. 56. The significant emotional impact, especially for community nurses, needs to be addressed alongside promoting effective, collaborative, and mutually supportive team working that can recognise and quickly adapt to changing patient needs.
12.	Walshe, C., Garner, I., Dunleavy, L., Preston, N., Bradshaw, A., Cripps, R. L., ... & CovPall study team. (2021). Prohibit, protect, or adapt? The changing role of volunteers in palliative and hospice care services during the COVID-19 pandemic. A multinational survey (CovPall). medRxiv.	To understand volunteer deployment and activities within palliative care services, and to identify what may affect any changes in volunteer service provision, during the COVID-19 pandemic.	Multi-national online survey disseminated via key stakeholders to specialist palliative care services, completed by lead clinicians. Data collected on volunteer roles, deployment, and changes in volunteer engagement.	458 respondents: 277 UK, 85 rest of Europe, and 95 rest of the world.	57. Flexible deployment plans need to be developed that protect volunteers, whilst still enabling them to have a role supporting care. 58. Consideration needs to be given to widening the volunteer base away from those who may be considered to be most vulnerable to COVID-19.

### Data synthesis

As the implications and recommendations for policy and practice would be descriptive, a thematic summary and synthesis was undertaken ( Seers, 2012). This involved collating the identified recommendations under nine anticipatory themes, taken from a collaborative stakeholder knowledge synthesis ( MacArtney *et al.*, 2021). For some themes there were several recommendations and so sub-themes were identified. SLD led the synthesis of the recommendations, presenting initial drafts to all the co-authors for discussion. SLD and JM led the writing-up of findings, with input from co-authors.

## Results

### Eligible literature

**Figure 1.**
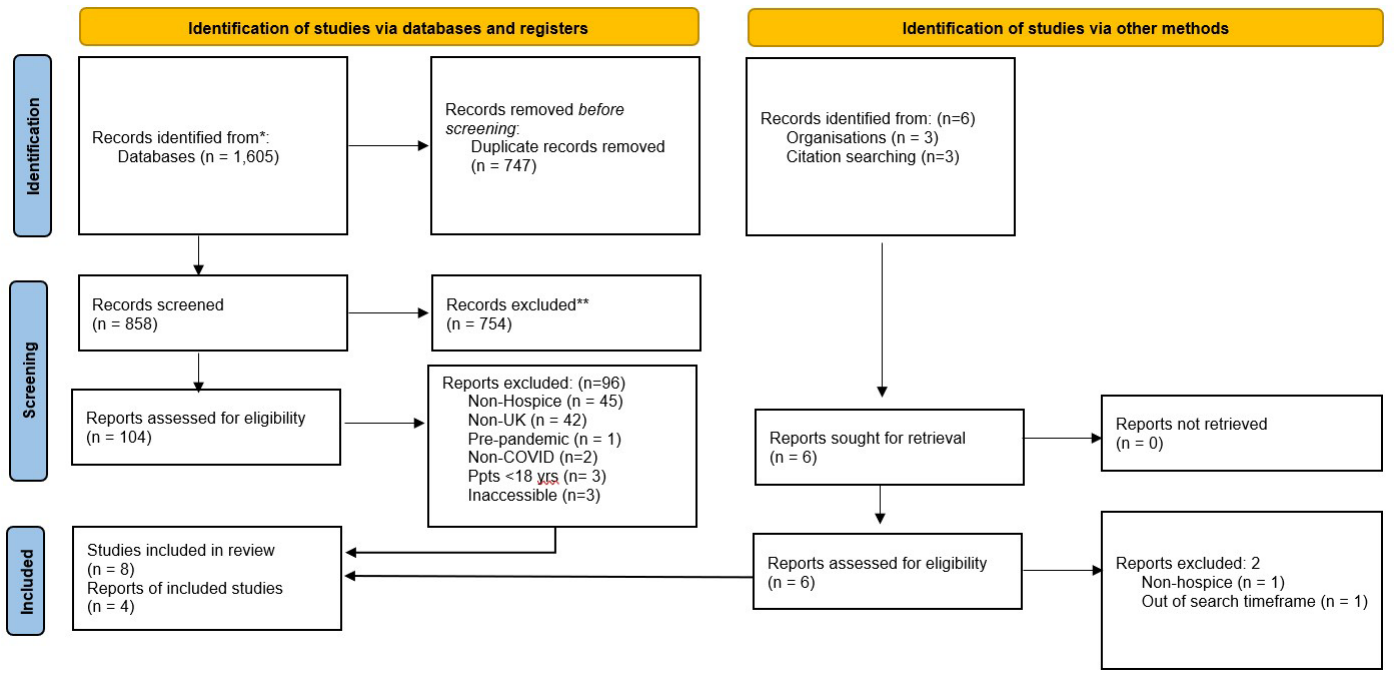
PRISMA flow diagram.

A PRISMA diagram of the screening process is provided ( Figure 1). The initial search produced 1,605 results, reduced to 858 identified once duplicates were removed, which were screened to identify 104 results. Titles, abstracts and, where necessary, full papers were then checked in detail for eligibility. In total, 12 outputs were included ( Table 2), with 8 articles included ( Bradshaw *et al.*, 2021; Hanna *et al.*, 2021; Mayland *et al.*, 2021; Mitchell *et al.*, 2021; Nestor *et al.,* 2021; Oluyase *et al.*, 2021; Pearce *et al.*, 2021; Walshe *et al.*, 2021) ( Table 2: 4, 6, 7, 8, 9, 10, 11, 12). Three further articles were found via citation searching ( Bayly *et al.*, 2022; Dunleavy *et al.*, 2021; Selman *et al.*, 2020) ( Table 2: 2, 3, 5). Following a search of grey evidence, one third sector report was also identified ( Marie Curie, 2021) ( Table 2: 1).

### Characteristics of included articles

One of the articles was a rapid evidence review (including systematic reviews and research studies, 5), nine of the studies were based on survey or online survey approaches (2, 3, 4, 6, 8, 9, 10, 11, 12), and the other an interview study (7). The sector report (1) incorporated evidence utilising all these approaches, as well as secondary analysis of research and publicly available data. The papers recommendations and implications were grouped into three sub-literatures: first those that seek to provide *snapshots and overviews of impact of Covid-19 pandemic on hospices* (1, 2, 3, 6, 9, 12); second, studies that explore *healthcare professionals providing end-of-life care during the first waves of the pandemic* (7, 8, 11); and, third, studies of *professionals providing bereavement care and relatives’ experiences* (4, 10).

***Sub-literature one: Snapshots and overviews of impact of Covid-19 pandemic on hospices.*** Five of the articles report findings from the CovPall study (2, 3, 6, 9, 12), as does the third sector report (1). The CovPall study presents findings from 458 services operating in the UK, Europe and the rest of the World (without further country details) and was based on an online survey methodology. Three of the five articles include findings from all of the 458 palliative care services (3, 6, 12). The other two articles reported the findings from the 61 rehabilitation leads for specialist palliative care services across hospice, hospital, or community settings (2), and the 277 UK services, of which 168 included hospice services (9). The latter included medical directors/lead medical clinician respondents (n=97, 35.4% of sample), nurse directors/lead nurse clinicians (n =69, 25.2% of sample) or other (n=108, 39.4% of sample).

In the UK arm of the CovPall study over half of the services (59.2%) provided care across 2+ settings; 48.7% hospital palliative care teams, 60.6% in-patient palliative care units and 57.8% home palliative care teams (n=277) (3, 9). Over half (54.6%) of the organisations were charitable organisations and 39.3% were public organisations (n=262). This is a particular contrast to the other data e.g. for Europe, the figure for charitable organisations is half that of the UK’s, at 27.1%, and similarly in Europe 60% of responses were from public-based organisations (n=85).

The Marie Curie Better End of Life report (1) draws on patient and carer perspectives (one member of the PPI reference group for the report and three carers), published literature, secondary analysis of research data and publicly available data – including the CovPall findings – to provide qualitative and quantitative perspectives in order to recommend improvements for end-of-life care. 

***Sub-literature two: Healthcare professionals providing end-of-life care during the first waves of the pandemic.*** The rapid evidence review synthesises evidence on what enables or hinders people in the community to make or update Advance Care Plans (ACP) in the context of Covid-19 and how staff working in health and social care can best support this process. The review included 21 research studies and 10 systematic reviews, plus 12 guidelines related to ACP in the UK during Covid-19 (5). The studies and reviews included findings from the range of stakeholders including palliative healthcare professionals (GPs, nurses, hospice and care staff), patients, and their families. The studies provide findings on ACP decision aids and interventions, communication in the context of ACP, and nurse-led post-discharge ACP.

The study by Mitchell *et al.* (2021) (11) looked at the views of GPs and community nurses providing end-of-life care, and was another web-based, UK-wide questionnaire survey circulated via professional general practice and community nursing networks during September and October 2020. In total, there were 559 respondents (387 community nurses, 156 GPs, and 16 unspecified roles) from all regions of the UK.

The only interview study in this review explored health and social care professionals’ experiences of providing end of life care during the Covid-19 pandemic (7). It included a range of professionals (n = 16), including registered nurses (n = 5); team leaders (nurse) (n = 1); clinical nurse specialists (n = 3); consultant clinicians (n = 2); junior doctors (n = 2); chaplain (n = 1); social worker (n = 1); and healthcare assistant (n = 1). The study aim was to help inform current/future clinical practice and policy, and participants had been recruited during the first pandemic wave, March-June 2020. The data was collected via telephone or video calls due to the nature of the pandemic restrictions on face-to-face interaction.

The study by Nestor *et al.* (2021) (8) aimed to describe the magnitude and variety of ways in which the Covid-19 pandemic affected the personal, social and professional lives of healthcare workers in a fully-integrated palliative and elderly care service. The study was survey based utilising an anonymised standardised questionnaire. In total, there were 250 respondents (69.8%), and nurses and healthcare assistants comprised the majority of respondents (n=150, 60%). The other staff groups were proportionately represented including ‘medical’ staff (n=15, 6%), administration staff (n=20, 8%), allied health professionals (n=17, 6.8%), and catering, household and maintenance staff (n=33, 13.2%), other (n=15, 6%).

***Sub-literature three: Professionals' and relatives' experiences of bereavement.*** One study investigated the experiences and views of practitioners in the UK and Ireland concerning changes in bereavement care during the Covid-19 pandemic, and thus linked to hospice care at the end of life in the respect of bereavement (4). This study was based on an online survey, and included a broad range of healthcare professionals (n=805): nurses (n=176, 22%), palliative care specialist nurses (n=103, 13%), community nurses (n=51, 6%), other nurses (n=22, 3%), bereavement counsellors, support workers or volunteers (n=173, 21%), chaplains (n=115, 14%), doctors (n=98, 12%), palliative care doctors (n=65, 8%), general practitioners (n=28, 3%), other doctors (n=5, less than 1%), those working in health and social care management (n=54, 7%), social workers or social care workers (n=52, 6%), allied health professionals (n=35, 4%), psychologists, psychotherapists and counsellors (n=30, 4%), bereavement service manager or coordinator (n=29, 4%), administration (n=27, 3%) and funeral directors/celebrants (n=19, 2%).

A second study explored bereaved relatives’ experiences of the care and family support provided during the last days of life (10). Participants were individuals (≥18 years) who had experienced the death of a relative/friend (all care settings) within the UK during the Covid-19 pandemic. The study, based on an online survey, included 278 respondents, and most (n = 216, 78%) were female. The survey was a national, open online survey disseminated via social media, public and professional networks during June–September 2020, thus incorporating the first six months of the pandemic period.

### Categorisation of themes and thematic synthesis

Fifty-eight recommendations or implications were identified, extracted and indexed ( Table 2). The recommendations were then sub-categorised by SLD as applicable to either Policy (n=31) or Clinical (n=27) recommendations, or both (n=10) ( Table 3). RD reviewed recommendations for Policy, and SM and CM reviewed those for Clinical. Policy was defined as “Policy, guidelines, or requirements that should be implemented through the executive”. Clinical included, “regulations which are or should be implemented through actions or decisions by health bodies, organisations or regulators or that can be implemented for better practice at individual clinical or service level”. This categorisation helped to identify the focus of recommendations and implications for practice, as well as areas where there were few or no implications reported. However, many recommendations were either so broadly designed or cross-cutting to be categorised across both. When summarising and synthesising the recommendations and implications we have sought to highlight, where possible, for whom or what level of health service delivery the recommendation may apply to. Recommendations are referred to in the Findings and Discussion as “Rx”.

**Table 3.** Categorisation of recommendations into anticipated themes.

Themes	Policy recommendations	Clinical recommendations
**Hospices: an overlooked** ** service**	***Recognition*** *For palliative services:* 2.Palliative care services have played a front-line role during COVID 19 and this role must be recognised 3.Societal preferences and expectation for death and dying have permanently changed and the health and social care system must respond accordingly 37. Palliative care services need equipment, medicines, and adequate staff to contribute fully to the pandemic response. Their crucial role must be better recognised and integrated, including into infection disease management, with improved workforce planning and management, so that patients and families can be better supported. 38.The crucial role of palliative care during pandemics must be better recognised and integrated. This is particularly the case for charity managed services and those providing care in people’s homes. *Recognition for palliative community services* 52.There is an immediate need for policymakers and commissioners to recognise the sustained increased need for end-of-life care in the community and the critical role of primary healthcare services in the delivery of this care. *For staff* 43. Need to recognise the importance of supporting all staff and keeping them safe by initiatives such as access to appropriate PPE; education, including support with adapting to greater use of IT; clear communication strategies, accurate and consistent information; staff involvement in protocol development and implementation, introduction of innovative means of communication with family members and colleagues *For patients, (carers and clinicians)* 36. Despite actively supporting dying patients, those with severe symptoms, their families/carers, other supporting clinicians, palliative care professionals felt ignored by national health systems during the COVID-19 pandemic. 5. Family members and carers must be recognised as important providers of palliative and end of life care and supported *Recognition for care homes:* 4. Care homes must be recognised as important providers of palliative and end of life care and supported appropriately *For primary care services:* 6. Primary care services need to be recognised as increasingly important providers of palliative and end of life care, at home and in care homes	
**Impact on resources and** ** funding**	*Resources* 1.Specialist palliative care services within and outside of the NHS must be resourced appropriately 13. In addition to financial support, greater collaboration is essential to build organisational resilience and drive forward innovation, by minimising duplication of effort and optimising resource use. 37. Palliative care services need equipment, medicines, and adequate staff to contribute fully to the pandemic response. Their crucial role must be better recognised and integrated, including into infection disease management, with improved workforce planning and management, so that patients and families can be better supported. 43. Need to recognise the importance of supporting all staff and keeping them safe by initiatives such as access to appropriate PPE; education, including support with adapting to greater use of IT; clear communication strategies, accurate and consistent information; staff involvement in protocol development and implementation, introduction of innovative means of communication with family members and colleagues *Training* 39. Beyond COVID-19, this research has shed light on the limited integration of palliative care, the urgent need to increase its workforce and a need for palliative skills to be a core part of the training of clinicians *Workforce* 37. Palliative care services need equipment, medicines, and adequate staff to contribute fully to the pandemic response. Their crucial role must be better recognised and integrated, including into infection disease management, with improved workforce planning and management, so that patients and families can be better supported. 39. Beyond COVID-19, this research has shed light on the limited integration of palliative care, the urgent need to increase its workforce and a need for palliative skills to be a core part of the training of clinicians *Finance /Funding* 37. Palliative care services need equipment, medicines, and adequate staff to contribute fully to the pandemic response. Their crucial role must be better recognised and integrated, including into infection disease management, with improved workforce planning and management, so that patients and families can be better supported.	13. In addition to financial support, greater collaboration is essential in relation to training, policies, and guideline development to build organisational resilience and drive forward innovation, by minimising duplication of effort and optimising resource use. 37. Palliative care services need equipment, medicines, and adequate staff to contribute fully to the pandemic response. Their crucial role must be better recognised and integrated, including into infection disease management, with improved workforce planning and management, so that patients and families can be better supported. 39. Beyond COVID-19, this research has shed light on the limited integration of palliative care, the urgent need to increase its workforce and a need for palliative skills to be a core part of the training of clinicians 47.Providing staff training and enabling protected time for timely, informative communication between health and social care professionals and family members needs to be prioritised during a pandemic, especially within the care home setting where this is less commonplace.
**Loss of volunteers**	57. Flexible deployment plans need to be developed that protect volunteers, whilst still enabling them to have a role supporting care. 58. Consideration needs to be given to widening the volunteer base away from those who may be considered to be most vulnerable to COVID-19.	
**Changes to visiting ** **arrangements**	41. Clarity in guidance and governance is required to identify when relatives can visit a dying family member in institutional settings during a pandemic, with a clear recommendation that this contact should be facilitated when death is expected in weeks and days rather than hour(s) before death.	40. Tools such as question prompt lists and charting daily family communication could help promote informative family engagement at times of restricted visiting.
**Impact upon the quality** ** of hospice care**		
**Demographies and** ** geographies of care**	*Access to benefits and services* 10.The UK government should work with local authorities to introduce a fast-track passport for people who have a diagnosed terminal illness that entitles them to access all relevant benefits and services on a fast-track or priority basis *Rehabilitation services* 11. Recommendations are made to support extended reach and more equitable access to rehabilitation in palliative care services.	
**Places of care: towards ** **integrated hospice care in** ** the community**	*Integration of palliative care* 13. In addition to financial support, greater collaboration is essential to build organisational resilience and drive forward innovation, by minimising duplication of effort and optimising resource use. 14. The effectiveness and sustainability of any changes made during the crisis needs further evaluation 38.The crucial role of palliative care during pandemics must be better recognised and integrated. This is particularly the case for charity managed services and those providing care in people’s homes. 39. Beyond COVID-19, this research has shed light on the limited integration of palliative care, the urgent need to increase its workforce and a need for palliative skills to be a core part of the training of clinicians 53. Findings suggest a disconnect between teams involved in end-of-life care in the community and a need to rebuild trusted relationships through truly integrated approaches between GPs, community nurses, and specialist palliative care services. 54. Policy guidance and service models must place focus on and support the multidisciplinary team relationships that are necessary to deliver this care most effectively. Current guidance relating to the roles of specific services has the potential to fragment teams.	
**Hospice at home**		(47. Providing staff training and enabling protected time for timely, informative communication between health and social care professionals and family members needs to be prioritised during a pandemic, especially within the care home setting where this is less commonplace. )
**Digital and remote** ** palliative healthcare**	8.Data systems must include information on dying death and bereavement	8.Data systems must include information on dying death and bereavement
**Changes to services that ** **worked, changes that did not** ** work**	Advance Care planning (case study) *Resources for ACP* 22.In the context of COVID-19, and to reduce inequalities in access to Advance Care Planning (ACP), we recommend national investment in evidence-based, public- facing resources and integrated systems to support ACP, building on existing resources. 45. Providers and policymakers need to urgently consider how high-quality advance care planning can be resourced and normalised as a part of standard care across the health sector, ahead of future or recurrent pandemic waves and in routine care more generally. *Guidance and policy to support and integrate ACP* 23.Alongside this investment, simultaneous, interconnected strategies are needed, underpinned by healthcare policy: training for those working in health and social care, better coordination of electronic medical record systems, and public education and awareness raising. 31. Targeting multiple levels of influence (individual, interpersonal, provider, system) to ensure ACP interventions are effective, sustainable and have maximum reach during the pandemic. At present most ACP guidelines focus on clinicians. 34. Ensuring each country in the UK has a comprehensive policy to support ACP and aid its implementation, monitoring and evaluation. 44. COVID-19 has provided an opportunity to re-think advance care planning in which the starting point to any discussion is always the values and priorities of patients themselves. 46.There are several key questions that health professionals, services, and policy makers ought to consider in working towards this. *Better use of digital technology* 23.Alongside this investment, simultaneous, interconnected strategies are needed, underpinned by healthcare policy: training for those working in health and social care, better coordination of electronic medical record systems, and public education and awareness raising. 33.Creating a robust, nationally co-ordinated, and public-facing web portal for ACP resources to facilitate this shift and increase awareness and uptake, harnessing the increased use of technological approaches to care and communication during the pandemic. It is essential that resources are diverse, use audio-visual as well as written formats, and are designed to support disadvantaged communities. 35. Prioritising research into an integrated web-based system for ACP in which members of the public could create an advance care plan which links to their medical record. *Public engagement about ACP* 24. Informing the public about the processes and legal status of ACP and dispelling fears and misperceptions, e.g., that ACP is related to rationing healthcare resources. 32.Introducing coordinated and consistent public health messaging that reframes ACP as routine and normal for anyone interested in considering and influencing their future care, making ACP driven by the public and supported (rather than owned) by health and social care professionals. 33.Creating a robust, nationally co-ordinated, and public-facing web portal for ACP resources to facilitate this shift and increase awareness and uptake, harnessing the increased use of technological approaches to care and communication during the pandemic. It is essential that resources are diverse, use audio-visual as well as written formats, and are designed to support disadvantaged communities.	*Resources for ACP* *Guidance and policy to support and integrate ACP* 23.Alongside this investment, simultaneous, interconnected strategies are needed, underpinned by healthcare policy: training for those working in health and social care, better coordination of electronic medical record systems, and public education and awareness raising. 25.Creating opportunities for ACP conversations among patients and residents early, particularly among older people and those at increased risk, discussing ACP over several sessions and revisiting decisions. 27.Adapting ACP to the individual and, if appropriate, including the opportunity to complete ACP documentation, without focusing on this. 44. COVID-19 has provided an opportunity to re-think advance care planning in which the starting point to any discussion is always the values and priorities of patients themselves. *Better use of digital technology* 28.Using remote consultations for ACP discussions where needed and appropriate, drawing on best practice guidelines. 30.Ensuring ACP discussions are fully and promptly recorded in patient records which are accessible to those who need them. *Public engagement about ACP* 24.Informing the public about the processes and legal status of ACP and dispelling fears and misperceptions, e.g., that ACP is related to rationing healthcare resources. 26.Sign-posting to appropriate written, web-based and audio-visual ACP resources. 29.Helping to cultivate a culture of openness around ACP in nursing home settings and having ongoing ACP conversations with residents (including those with cognitive impairment) and their family members. 32.Introducing coordinated and consistent public health messaging that reframes ACP as routine and normal for anyone interested in considering and influencing their future care, making ACP driven by the public and supported (rather than owned) by health and social care professionals. Staff well-being (case-study) 21.Acknowledging the challenges on staff and encourage bereavement training for those who feel unequipped to manage needs of grieving families. 42. There is a need for visible leadership and support within healthcare teams to promote self- care and reflection, as well as ongoing access to psychological support for health and social care professionals. 48. During a pandemic, it is essential that health and social care staff can recognise dying and feel confident to talk honestly with relatives about this, to enable final visits to be conducted in a timely manner. 55.Ensuring support for individuals involved in the provision of this care, through team relationships, training opportunities, and debrief also requires attention. 56.The significant emotional impact, especially for community nurses, needs to be addressed alongside promoting effective, collaborative, and mutually supportive teamworking that can recognise and quickly adapt to changing patient needs.
**Impact on bereavement** ** support**	*Support for bereavement services* 7. Increased provision of bereavement services is urgently needed 15. Improved resources for existing bereavement services to enable coordination between local, regional and national networks, and encourage a sustainable model of bereavement care. 16. Develop a proactive approach to supporting those bereaved during this period and making services accessible for all. Coordination of bereavement services (local, regional, national) *Resources for bereavement services* 15. Improved resources for existing bereavement services to enable coordination between local, regional and national networks, and encourage a sustainable model of bereavement care	15.Improved resources for existing bereavement services to enable coordination between local, regional and national networks, and encourage a sustainable model of bereavement care. 16.Develop a proactive approach to supporting those bereaved during this period and making services accessible for all. 17.Enable regular communication with families prior to a death and after to ensure families have opportunities to ask questions and receive reassurance. 18.Where possible, find ways for families to be with dying loved ones. 19.Integrating assessment of bereaved families’ needs into communication to help identify and signpost those who might require further support. 20.Training in bereavement care to be integrated into medical, nursing, and other healthcare professional training. 21.Acknowledging the challenges on staff and encourage brief training for those who feel unequipped to manage needs of grieving families.

**Hospices: An overlooked service**

There were nine policy recommendations or implications seeking *recognition* of hospice palliative care services (Rs 2, 3, 4, 5, 6, 36, 37, 38, 43, 52). This included the need to recognise the “front-line” (R2) and “crucial” (Rs 37, 38) role all palliative care services played in a range of locations – hospice, care homes, hospital, primary care, community and people’s homes – during the pandemic (Rs 4, 6, 38, 52). It was recommended that ways be found to hear the voices of palliative care patients, carers and professionals – all of whom had been found to feel largely overlooked by the NHS during the Covid-19 pandemic (R36). As well as reporting on findings from those affected, two papers suggested recognition could be evidenced via better funding and integration of palliative care services both into the health service more generally and specifically during the pandemic (R37, 38, 52), with one highlighting the need for better workforce planning and management, so that patients and their families can be better supported (R38). It was also noted that recognition of palliative care staff could be demonstrated through better access to PPE, education, clear communication strategies between managers and staff, accurate and reliable information, and new ways of communication with family members and colleagues (R43).

***Impact on resources.*** There were six recommendations or implications related to the need for palliative care services to be resourced appropriately (Rs 1, 3, 13, 15, 37, 43). It was stated that, “societal preferences and expectations for dying, death and bereavement have been permanently changed” by the pandemic and that the health and social care system will need to respond accordingly – both during the pandemic and after (R3: p5). Three recommendations specified the types of resources which are crucial for palliative care to work more effectively during a pandemic, including equipment, medicines, effective communication, access to PPE, adequate staff education and training, and ensuring staff had protected time for updates and inter-service communication (Rs 37, 43, 47). An increased palliative care workforce was also recommended as crucial to improving future palliative care delivery (Rs 37, 39), with training in palliative care recommended for all clinicians (R39).

There were two recommendations that were concerned with hospice palliative care’s wider funding structures. It was recognised that specialist palliative care services had been flexible and had adapted to the exceptional circumstance of the pandemic with low-cost solutions (3). However, there were two recommendations that the sector is funded more effectively in the future to provide better palliative and end-of-life care (Rs 13, 37)

***Volunteer role.*** Two recommendations focused on the volunteer workforce, so crucial to the running of many hospices, but who were not often seen as a staffing priority during the pandemic. It was recommended that more flexible arrangements could be made that would help protect volunteers during a pandemic and so enable them to volunteer (R57). It was also suggested that hospices diversify the demographic base of volunteers, so it is less dependent upon those that might be vulnerable to Covid-19 (R58).

***Visitation.*** There was only one paper that focused on hospice visiting (7), which urged hospices to be clearer about when relatives can visit a dying family member during a pandemic and that this communication should take place in the weeks and days, rather than hours, before death (R41). To help facilitate this, it was recommended that clinicians use question prompt lists, as well as ensure communication with the family is properly documented, especially at times of restricted visiting (R40).

**Impact upon the quality of hospice care**

***Demographies and geographies of care.*** There were two recommendations that focused on better access to services. The first recommends the government should introduce a fast-track passport for people who are diagnosed as terminally ill, enabling them to access all relevant benefits and services on a priority basis (10). The second focused on hospice-based rehabilitation services, but is generalisable to all hospice care, and recommended more equitable access to palliative care (rehabilitation) services (11).

***Integration of palliative care.*** There were six recommendations on the need for increased integration of palliative care (Rs 13, 14, 38, 39, 53, 54). Two papers found that hospices – particularly charity funded – and specialist palliative care in the community were not always well integrated with primary care, community nurses, or the national health system in general (6, 11). It was argued that there is an urgent need to recognise the need for palliative care in pandemics, increase numbers of the specialist palliative care workforce, and ensure palliative care skills are part of the training of general clinicians (Rs 38, 39, 53). The focus of some guidance on the specific roles of particular services was seen to contribute to the fragmentation of services and it was recommended that greater emphasis be placed on multidisciplinary teams and (re)building trust between services (Rs 53, 54). It was recommended that greater collaboration across national and international settings should take place to optimise resource use and avoid duplication of effort, while maintaining high standards of care, particularly in relation to training, policy and guidelines development (R13). Importantly, it was recognised that the effectiveness and sustainability of any changes made during the pandemic (and afterward) will need evaluating (R14).

***Hospice at home.*** There were no recommendations that specifically addressed the provision of ‘hospice at home’, although several discussed elsewhere (such as those on ACP e.g. Rs 22-30; digital technology e.g. R33; and resources and funding e.g. Rs 1, 13, 37, 39; and training e.g. R47 and others) are relevant to developing a ‘hospice at home’ service during the pandemic.

***Digital and remote palliative healthcare.*** Digital technologies to facilitate remote palliative care were expected to be an important part of hospices’ pandemic response ( MacArtney *et al.*, 2021). However, there were only two recommendations that directly addressed this issue. The first, the Better End of Life report by Marie Curie (1), recommended that data systems must include information on dying, death and bereavement (R8). The second was focused on the facilitation of better Advance Care Planning (ACP, discussed in detail below), and recommended that a “robust, nationally co-ordinated, and public-facing web portal for ACP resources” be developed to help improve communication both during the pandemic and after (R33).

***Changes to services that worked, changes that did not work.*** It was expected that there would be case-studies, specific moments or issues during the pandemic that may provide learning opportunities ( MacArtney *et al.*, 2021). For this review, two sets of recommendations were evident, one relating to Advance (or ‘Anticipatory’, in Scotland) Care Planning (ACP), and the other concerned hospice and specialist palliative care staff wellbeing.

Two sources were identified that specifically addressed the use of ACP in the pandemic: a rapid review (5); and the national CovPall survey of SPC clinicians, which included a large proportion of hospice staff (9). From these we identified 16 recommendations for ACP, which we organised under four sub-themes. 

Both the review and survey highlighted the need for national initiatives to *fund ACP resources*, integrate systems, and reduce inequalities of access to ACP within palliative care contexts and across all healthcare services (Rs 22, 45). It was recognised that the pandemic brought an opportunity to develop *guidance and policy to support and integrate ACP* across the UK (Rs 23, 34, 35). This included refreshing understandings of ACP as starting with the patient’s values and priorities, be undertaken early and often, and that processes should be adapted to individual needs (Rs 25, 27, 44). Better implementation of ACP will involve training at multiple levels (e.g. health professionals, interpersonal, service providers, system, and/or policy makers), across all sites of healthcare, including care homes, with “key questions” being identified for each level to reflect upon (Rs 25, 31, 46). To support integration of ACP, several ways to make *better use of digital technology* were identified, including use it for remote consultations (R28), recording of conversations in patients’ electronic notes (R30), coordination of IT systems so medical records and ACP can be shared across healthcare services (R23); a public-facing web portal of ACP resources (Rs 33, 35); and use of multiple digital media to further *public engagement with ACP*, particularly with disadvantaged communities (Rs 26, 33). A consistent public engagement strategy was also identified as necessary to create a sense of openness, public ownership, normalise planning, and dispel fears of ACP, especially in care home settings (R24, 29, 32).

The impact of the pandemic on hospices was also recognised to effect staff wellbeing, as well as the services they provide. Recommendations focused on what employers, managers and colleagues can do for each other to promote self-care, provide psychological and emotional support, develop and maintain relationships, find time to debrief and reflect, access training to assist in managing own and families’ bereavement and grief, and to support staff during periods of rapid service change and uncertainty (Rs 21, 42, 48, 55, 56).

***Impact on bereavement support.*** Seven recommendations focused on bereavement services drawing urgency from the need to increase the provision of bereavement services due to the pandemic (R7). This involves improving resources for existing bereavement services (R15), and ensuring coordination between local, regional and national bereavement services and networks to provide training to all healthcare staff, so that they proactively work together and communicate with families – before and after a death – to ensure that those who may need specialist bereavement support are identified and referred (Rs 16, 17, 18, 19, 20, 21).

## Discussion

The Covid-19 pandemic and protections has highlighted several ongoing policy and practice needs, especially around hospice resources, as well as generating new issues for hospices to address. We found that significant policy gaps remain to be addressed to mitigate the impact of the pandemic on the quality of hospice specialist palliative care. Given the unique context of Covid-19, the public health protections put in place and the timeframe of the review, it was unsurprising to see that there were numerous recommendations addressing the impact of the pandemic and protections upon hospice resources. These recommendations addressed significant issues for maintaining hospice services including finances, clinical and physical infrastructure (access to buildings, equipment, medicines, PPE etc), and staff workforce, training and wellbeing. That these were brought into such considerable question highlights how, in this context, hospices’ independent charitable status became doubly problematic: first, underscoring the degrees of operational and financial separation from mainstream state-funded provision that hospices in the UK experience (1); and, second, the precarious nature of the charitable funding model of healthcare ( Hospice UK, 2017).

The recommendations addressing the impact of the pandemic on the quality of hospice care were largely focused on three themes. First, the pandemic and protections highlighted a long-standing drive for hospice-based specialist palliative care to be better integrated with other services in the community, including primary care ( Gomez-Batiste *et al.*, 2017; WHO, 2018). Second, the role that hospices could take in leading and providing bereavement support was another prominent theme, with several recommendations intersecting calls for better integration and increased resources. Third, were the recommendations focused on how best to implement Advance Care Plans (ACP) in the pandemic context (5, 9).

### Limitations of the review

The review included studies that merged hospice data and evidence with wider palliative care service discussions, which meant that some recommendations were generalised from non-hospice palliative care settings to the hospice context. Where the papers reviewed made it possible, we have sought to highlight this generalisation in our synthesis preferring to include potentially useful, applicable recommendations than exclude them for any perceived ambiguity of origin or potential application. Ten papers provided recommendations building on primary research, but only two (7, 10) did not draw on staff survey data. There is therefore a significant lack of lived experiences, especially of patients, but of those that care for them, and hospice staff; key perspectives that are needed to develop a contextual richness to recommendations to help improve policy and practice.

The review has identified recommendations for practice and policy from papers published in the first 24 months of the pandemic. However, given the time lapse between data collection and publication, the recommendations are largely based on evidence collected in the first 12 months. Although the recommendations were generated during the pandemic, many are likely to remain relevant beyond this period e.g. recommendations relating to ACP (Rs 22, 23, 25, 27, 31, 34, 44, 46); digital healthcare (Rs 8, 23), and bereavement services (Rs 7, 15-21). However, some recommendations were specific to the needs of services during ‘lockdowns’ and during periods when higher levels of prevention and protection measures were in place e.g. around pandemic related need for increased resources (Rs 36, 37, 38); or visiting (R41). Several recommendations that looked to longer term implications can be seen to frame the pandemic as highlighting or exacerbating pre-existing issues and so were an opportunity to restate demands, such as for more secure funding (Rs 1, 13) and integrating palliative care more widely across healthcare services (Rs 13, 39).

We have not evaluated the quality of the studies that the recommendations came from or provided an evaluation of individual recommendations. Nor have we been able to conduct an ethnicity impact assessment of policy recommendations ( Bajwah *et al.*, 2021). Our aim was to summarise and synthesise the multiple recommendations produced by numerous investigations and reports during the pandemic. As one study noted, further research and evaluation will be needed to gauge whether a recommendation can be (or has been) successfully implemented ( Dunleavy *et al.*, 2021).

### Implications for future research and policy

We structured our analysis around the themes identified as important by of academic experts, clinical stakeholders, and PPI representatives as part of a collaborative stakeholder knowledge synthesis ( MacArtney *et al.*, 2021). However, this review has also shown that there were relatively few recommendations addressing several key areas previously found to be important for adapting hospice services to the pandemic or post-pandemic future. For example, while there was much discussion in social and traditional media of the difficulties of visiting relatives in health and social institutions ( Joint Committee on Human Rights, 2021), only one study had provided recommendations relating to the specific visiting issues faced by hospices (7). Similarly, there was only one recommendation (R22) that sought to attend to the inequitable impact of the pandemic upon hospice service users, and this was specifically related to accessing ACP. There were no recommendations that considered the impact of the pandemic on different demographic groups or how the recommendations may affect those groups differently and equitably. Lastly, there were a lack of recommendations addressing the rapidly growing need for hospice-at-home; something that needs to be addressed as more people are expected to request to have their final weeks at home ( Bone *et al.*, 2018; Rees-Roberts *et al.*, 2019).

## Conclusion

The impact of the pandemic and protections further exposed or accelerated many issues known about in hospice services pre-pandemic. While many recommendations for policy and practice echo these pre-existing needs and requests, several pandemic specific recommendations were identified, although these were often more limited in scope due to the novelty of the Covid-19 situation. What this review has shown is that there even after two years of action, research and review, there are significant gaps in knowing what needs to be done or changed to address the impact of Covid-19 upon key areas of hospice services and provision of care. As Covid-19 continues to circulate in the wider population the needs of patients, carers and staff will change and with it most, if not all, previous recommendations may need adapting and refinement over time.

## Registration and protocol

A protocol was prepared but not registered.

## Data Availability

All data underlying the results are available as part of the article and no additional source data are required. Zenodo: PRISMA Checklist for ‘ *The impact of Covid-19 pandemic on hospices: A systematic integrated review and synthesis of recommendations for policy and practice’*, https://doi.org/10.5281/zenodo.7157181 ( MacArtney, 2022). Data are available under the terms of the Creative Commons Attribution 4.0 International license (CC-BY 4.0).
