# Supplementary material for: The impact of Covid-19 pandemic on hospices: A systematic integrated review and synthesis of recommendations for policy and practice
Source: AMRC Open Res. 2022 Oct 26;4:23. [Version 1] doi: 10.12688/amrcopenres.13105.1 (PMC11064931; doi:10.12688/amrcopenres.13105.1)
Supplement: Peer review history

# Reviewer report: Reviewer response for version 1

**DOI:** 10.21956/amrcopenres.14184.r27017

**Published:** 2022-11-15

**Authors:** Glenys Caswell

**Affiliations:** 1 School of Health Sciences, University of Nottingham, Nottingham, UK

This review presents a synthesis of recommendations and implications for both policy and practice for hospices in the United Kingdom. The literature drawn on was published during the first two years of the covid19 pandemic. The rationale for this is clearly laid out, as providers of palliative care within the hospice sector were impacted by the demands for care resulting from the pandemic and also by the restrictions imposed and the need to identify different ways of working.

 The authors searched a good range of databases and identified more than 800 papers, which were whittled down to 12. The process by which this happened and the roles of the different members of the team are clearly delineated. Each of the papers which made the final cut is described. Review of the cited papers resulted in 58 recommendations and/or implications for practice and policy. The authors have presented these separately and as encapsulated within 10 themes. They are presented in list form as well as described. Some of the themes and recommendations are, inevitably, specific to the endeavour to provide a service during a pandemic, but there are others, such as sources of funding within the hospice sector, that are an ongoing issue.

 This is a useful piece of work, pulling together published work and demonstrating what has been examined and what still requires to be examined in the field. The impact on hospice service users, for example, and the increasing need for hospice at home services are topics which were not well covered by the literature.

 I would recommend that the authors give the paper a final proofread. I noted a small number of typographical errors, for example in the abstract where it reads ‘searchers’ instead of ‘searches’.

Are the rationale for, and objectives of, the Systematic Review clearly stated?

Yes

Is the statistical analysis and its interpretation appropriate?

Not applicable

Are sufficient details of the methods and analysis provided to allow replication by others?

Yes

Are the conclusions drawn adequately supported by the results presented in the review?

Yes

Reviewer Expertise:

Sociologist researching issues pertaining to palliative and end of life care

I confirm that I have read this submission and believe that I have an appropriate level of expertise to confirm that it is of an acceptable scientific standard.

# Response

**Published:** 2022-11-22

**Authors:** John MacArtney

**Affiliations:** University of Warwick, UK

Thank you Dr Glenys Caswell for your supportive review. We have proof read the manuscript and believe we have identified the remaining typos.

# Reviewer report: Reviewer response for version 1

**DOI:** 10.21956/amrcopenres.14184.r27016

**Published:** 2022-11-15

**Authors:** Tessa Morgan

**Affiliations:** 1 School of Nursing, University of Auckland, Auckland, New Zealand; 2 Department of Public Health and Primary Care, University of Cambridge, Cambridge, UK

Thank you for the opportunity to review this manuscript. It provides a useful and timely summary of changes to hospice policies during the first year of the pandemic. Emphasising issues around funding and service provision in this period should be relevant to both service providers and policy makers going forward.

 Please find below minor comments:

 Appropriate search strategy combining database and hand-searching to capture grey literature.

 Useful characterisation of themes. I wonder if it should be stated earlier in the results around how there is no specific mention of equity in these policies. A sense of the geographic spread of the policies.

 There is no quality appraisal of policies, but the authors adequately justify this as the aim is to summarise and synthesis the literature.

 I did want to know more about the extent to which there contradictions/conflicting views across the included policies? Did this appear to be region based/service based?

 I wondered about the inclusion of qualitative study. Does it fit with the remit of this paper around synthesising recommendations and implications for policy and practice. Might want one line around why it does.

 The inclusion of the theme around changes specifically identifying what did not work could have been addressed more directly. What exactly did not work? This would be important to clarify before suggesting what could be improved.

Are the rationale for, and objectives of, the Systematic Review clearly stated?

Yes

Is the statistical analysis and its interpretation appropriate?

Not applicable

Are sufficient details of the methods and analysis provided to allow replication by others?

Yes

Are the conclusions drawn adequately supported by the results presented in the review?

Yes

Reviewer Expertise:

Palliative Care, especially family/ informal caregiving. Interest in COVID-19 research as it relates to older people's experiences. Substantial experience with publishing systematic reviews.

I confirm that I have read this submission and believe that I have an appropriate level of expertise to confirm that it is of an acceptable scientific standard.

# Response

**Published:** 2022-11-22

**Authors:** John MacArtney

**Affiliations:** University of Warwick, UK

Thank you Dr Tessa Morgan for taking the time to provide such helpful comments on our article. We have responded to your specific comments below and included amendments in Version 2.  

*I wonder if it should be stated earlier in the results around how there is no specific mention of equity in these policies. A sense of the geographic spread of the policies.*

 Thank you for highlighting this issue. We checked the recommendations again for mentions of equity or inequitable access and noted one (R22) should have been included in “Demographies and geographies of care”. We have amended the paragraph as follows, also including a sentence to highlight earlier the paucity of recommendations on equity of access and to note the lack of geographic consideration in policy recommendations, “There were three recommendations that focused on better access to services. The first recommends the government should introduce a fast-track passport for people who are diagnosed as terminally ill, enabling them to access all relevant benefits and services on a priority basis (R10). The second sought to reduce inequalities in accessing Advanced Care Planning (ACP), recommending investment in resources and systems to support delivery of ACP (R22). The third focused on hospice-based rehabilitation services, but is generalisable to all hospice care, and recommended more equitable access to palliative care (rehabilitation) services (R11). The latter two were the only recommendations to specifically address the equitable provision of services. No recommendations addressed the varying palliative needs associated with different geographic locations (e.g. urban, suburban or rural).” To reflect this change to the Results, we amended the discussion to change “different” to “specific”, “There were no recommendations that considered the impact of the pandemic on *specific* demographic groups or how the recommendations may affect those groups differently and equitably.  

*I did want to know more about the extent to which there contradictions/conflicting views across the included policies? Did this appear to be region based/service based?*

 Thank you for raising this interesting line of enquiry. We agree that further comparison and evaluation would be helpful. However, developing the framework through or against which policy recommendations could be compared was beyond the scope and timeframe of this review.  

*I wondered about the inclusion of qualitative study. Does it fit with the remit of this paper around synthesising recommendations and implications for policy and practice. Might want one line around why it does.*

 We have provided some emphasis as to why we included this qualitative paper on p24, sub-section “Sub-literature two: Healthcare professionals providing end-of-life care during the first waves of the pandemic”:

 “ *The paper was included in this review, as* the study aim was to help inform current/future clinical practice and policy, and participants had been recruited during the first pandemic wave, March-June 2020.”  

*The inclusion of the theme around changes specifically identifying what did not work could have been addressed more directly. What exactly did not work? This would be important to clarify before suggesting what could be improved.*

 We agree that reflecting on what did not work will be necessary to developing better recommendations in the future. Perhaps due to the positive and interventionist nature of policy recommendations, none were framed deontologically i.e. “we recommend that you do not do X”. Again, it was beyond the scope of this review to explore in detail the basis for a recommendation. But future reviews may want to evaluate the findings behind the recommendations to elucidate the rationale around why they are framed as they are, including identifying and failed or partial policy successes
